# Phenotype Refinement Influences GWAS‐Implicated Variant Effect Sizes for Insomnia

**DOI:** 10.1111/jsr.70356

**Published:** 2026-05-26

**Authors:** Amber J. Zimmerman, Patrick Z. Liu, Brendan T. Keenan, Thomas G. Brooks, Struan F. A. Grant, Philip R. Gehrman, Ron C. Anafi, Allan I. Pack

**Affiliations:** ^1^ Division of Sleep Medicine, Department of Medicine, Perelman School of Medicine University of Pennsylvania Philadelphia Pennsylvania USA; ^2^ Department of Genetics, Perelman School of Medicine University of Pennsylvania Philadelphia Pennsylvania USA; ^3^ Divisions of Human Genetics and Diabetes & Endocrinology Children's Hospital of Philadelphia Philadelphia Pennsylvania USA; ^4^ Center for Spatial and Functional Genomics Children's Hospital of Philadelphia Philadelphia Pennsylvania USA; ^5^ Institute for Translational Medicine and Therapeutics, Perelman School of Medicine University of Pennsylvania Philadelphia Pennsylvania USA; ^6^ Department of Pediatrics, Perelman School of Medicine University of Pennsylvania Philadelphia Pennsylvania USA; ^7^ Department of Psychiatry, Perelman School of Medicine University of Pennsylvania Philadelphia Pennsylvania USA

**Keywords:** BTBD9, GWAS, insomnia, MEIS1, RLS, UK biobank

## Abstract

How phenotypes are measured, especially when relying on subjective reports, is an impediment to the utility of genome‐wide association studies (GWAS). This is a common problem in GWAS for sleep traits, as many sleep disturbances appear subjectively similar despite having distinct underlying pathophysiology. Phenotype refinement is, therefore, necessary to improve our understanding of complex trait biology. Here, we utilise expanded questionnaire data collected from ~180,000 participants in the UK Biobank to help further distinguish insomnia from restless legs syndrome (RLS) from subjective reports. We demonstrate prior GWAS efforts for insomnia likely mischaracterised participants using a single‐question approach. Through statistical models, we examine two of the most significant GWAS signals for insomnia, namely at the *MEIS1* and *BTBD9* loci*,* finding their effects are largely or completely, driven by their effects on RLS. Collectively, our results underscore the necessity of improving phenotype classification and highlight the utility of newly released UK Biobank data for sleep research.

## 1 Introduction

Genome‐wide association studies (GWAS) have revealed novel loci for complex traits in human populations. Exceptionally large GWAS have been performed for insomnia, the most common sleep disorder, using questionnaire‐based approaches performed within biobanks (Watanabe et al. 2022; Lane et al. 2017; Jansen et al. 2019; Hammerschlag et al. 2017). However, these analyses have historically been based on a single question, impairing our ability to specifically differentiate insomnia as an independent disorder from insomnia secondary to comorbid or phenotypically similar sleep disorders. In the latter case, GWAS signals may be driven by a comorbid disorder rather than primarily insomnia per se. Researchers have, therefore, sought better phenotyping to resolve potential confounding in insomnia GWAS reports (Watanabe et al. 2022; Jansen et al. 2019; Buysse et al. 2006; Gehrman et al. 2013; El Gewely et al. 2018).

GWAS efforts for insomnia, restless legs syndrome (RLS), and periodic leg movements in sleep (PLMS) have identified several common genetic variants (Watanabe et al. 2022; Schormair et al. 2024; Edelson et al. 2023). There has been substantial debate as to whether these associations are due to true underlying pleiotropy or phenotypic mischaracterisation (Watanabe et al. 2022; El Gewely et al. 2018; Sarayloo et al. 2019; Leu et al. 2024). Two of the most significant associations identified from insomnia GWAS reports reside within the genes *MEIS1* and *BTBD9* (Watanabe et al. 2022 *;* Jansen et al. 2019). However, both of the lead variants reported for these loci are in strong linkage disequilibrium with the reported RLS and PLMS associated sentinel variants at these loci (Watanabe et al. 2022; El Gewely et al. 2018; Schormair et al. 2024; Didriksen et al. 2020). Thus, it remains unclear if these robust associations with insomnia are due to comorbid RLS.

To address this potential confounding, we leveraged the newly released responses to an expanded sleep questionnaire series administered to a large cohort (> 180,000 participants) within the UK Biobank. This study sought to utilise these new questionnaire data to better refine sleep phenotypes and evaluate controversial genetic associations previously reported for insomnia. In so doing, we illustrate the power of these data to improve the ability to identify disorder‐specific genetic associations in future GWAS.

## 2 Methods

### 2.1 UK Biobank Sleep Questionnaire Analysis

At the time of our data collection (December 2025), the UK Biobank provided data on 501,936 participants. Of these, 180,833 completed the online sleep questionnaire (Category ID 205) that includes the validated Sleep Condition Indicator (SCI) (Espie et al. 2014, 2018) questionnaire (Category ID 209) for defining insomnia based on DSM‐5 criteria (Table S1). Because our variants of interest have differing population allele frequencies, we filtered for European‐only participants, which excluded 19,573 participants.

To identify participants with RLS, we used responses to the Cambridge–Hopkins Restless Legs Syndrome Questionnaire (CH‐RLSq) (Allen et al. 2009) collected by the UK Biobank (Category ID 212). The CH‐RLSq identifies individuals with a high probability of RLS using a branching logic method. Specifically, participants were asked a prompt question that queries potential RLS. Individuals only answered subsequent questions associated with CH‐RLSq if they answered ‘yes’ to either RLS prompt question 1 (Field Code 30579) or 2 (Field Code 30580) in Table S2. Participants that did not complete the RLS questionnaire were not included in the RLS category. Criteria for defining RLS is presented in Table S2. Numbers of participants completing each questionnaire are presented in Supporting Information Data. The UK Biobank excluded question 7a of the CH‐RLSq and added the question ‘In the past 12 months, how often did you experience these feelings in your legs’? (Field Code 32070). To further refine the RLS category by severity, we utilised data on frequency to define intermittent RLS as ≤ 1*x* per week and persistent RLS as > 1*x* per week (Nichols et al. 2003), in addition to responses listed in Table S2.

We further grouped participants into four categories based on presence/absence of insomnia/RLS as follows: controls (SCI > 16 and no RLS), isolated insomnia or ‘insomnia only’ (SCI ≤ 16 and no RLS), isolated RLS or ‘RLS only’ (SCI > 16 and RLS with no frequency threshold) and ‘insomnia with RLS’ (SCI ≤ 16 and RLS with no frequency threshold). Additional refinement based on frequency was added by including ‘persistent RLS’ versus ‘intermittent RLS’ as described above. Prior to analysis, we removed shift workers (Field code: 30431) and individuals with OSA (ICD 10: G47.3). Finally, we excluded those with poor quality or no sequencing data. Numbers of participants and exclusions are provided in Supporting Information Data.

To compare response proportions between questionnaire‐based approaches, we obtained responses to the question used by previous insomnia GWAS (Data‐Field 1200) for each participant included in our instrumentally‐defined sub‐groups. Response options for DF‐1200 ‘Do you have trouble falling asleep at night or do you wake up in the middle of the night’? included ‘prefer not to answer’, ‘never/rarely’, ‘sometimes’ and ‘usually’. Response proportions are provided in Supporting Information Data.

Multinomial logistic regression models used categorical disorder status as an outcome and SNP [rs61192259(C) or rs113851554(T), coded additively with respect to the number of minor alleles] as a predictor. Categories included control, insomnia only, RLS only or insomnia with RLS; persistent/intermittent RLS was evaluated in additional models. Two adjusted analyses were performed: Model 1 adjusted for genotyping array and the first 10 genetic principal components to account for population stratification; Model 2 added age at time of questionnaire, sex and body mass index (BMI) to Model 1.

Additional logistic regression models defined insomnia status as a binary variable for ‘yes’ or ‘no’ and analyses were performed adjusted according to the same covariates as the multinomial models, as well as using an additional Model 3 that added RLS severity to Model 2 covariates. In these models, RLS severity was defined as intermittent (≤ 1*x* per week) or persistent (> 1*x* per week). RLS severity was coded as a factor in the order: none (0), intermittent (1), persistent (2).

Linear regression models tested SNP associations to the quantitative sleep score from the SCI. The SNP was again coded additively with respect to the number of minor alleles and multiple adjusted models were examined. Similar to the logistic regression models described above, Models 1 and 2 included the same covariates listed for the multinomial logistic regression models, while Model 3 added RLS severity to Model 2. For all models, *p*‐values were calculated using two‐sided Wald tests.

## 3 Results

### 3.1 Defining Groups Using Clinically‐Relevant Sleep Questionnaires in the UK Biobank

We first defined four categorical groups using questionnaires from the UK Biobank, based on validated instruments to define clinically relevant insomnia (SCI) (Espie et al. 2014, 2018) and RLS (Cambridge–Hopkins RLS questionnaire) (Allen et al. 2009) (Tables S1 and S2). A total of 137,363 participants were included in the final analyses and further subdivided into controls (*n* = 95,782 [69.7%]), ‘insomnia only’ (*n* = 30,530 [22.2%]), ‘insomnia with RLS’ (*n* = 4463 [3.2%]), and ‘RLS only’ (*n* = 6588 [4.8%]) (Supporting Information Data). When participants in the RLS group were further subdivided based on frequency, 2.2% of participants had persistent RLS (symptoms > 1 day/week) and 2.6% had intermittent RLS (≤ 1 day/week). This cohort of participants was older (mean [SD] age of 70.3 [7.4] years), overweight (BMI of 26.7 [4.5] kg/m^2^) and 58% were female. Demographics for each group are provided in Tables S3 and S4. Consistent with previous reports (Watanabe et al. 2022; Schormair et al. 2024), insomnia and RLS were more frequent in females in this cohort that completed the online sleep questionnaires.

### 3.2 Responses by Group to the Previous Single Question Used for Insomnia GWAS

As previous GWAS reports for insomnia using the UK Biobank data were based on the single question ‘Do you have trouble falling asleep at night or do you wake up in the middle of the night’? (Watanabe et al. 2022; Lane et al. 2017; Jansen et al. 2019; Hammerschlag et al. 2017), we compared the proportion of overlap between responses to this question and our sub‐groups defined using multi‐question validated instruments. A response of ‘usually’ to this single question was required for inclusion into the ‘insomnia’ category for previous GWAS; however, we observed 21.2% of controls, as defined by the expanded questionnaire and 31.2% of ‘RLS only’ individuals responding with ‘usually’ to this question. The proportions were higher in the ‘insomnia only’ (57.6%) and insomnia with RLS (63.8%) groups, but this single question does not sufficiently rule out RLS (Figure 1 and Table S5).

**FIGURE 1:**
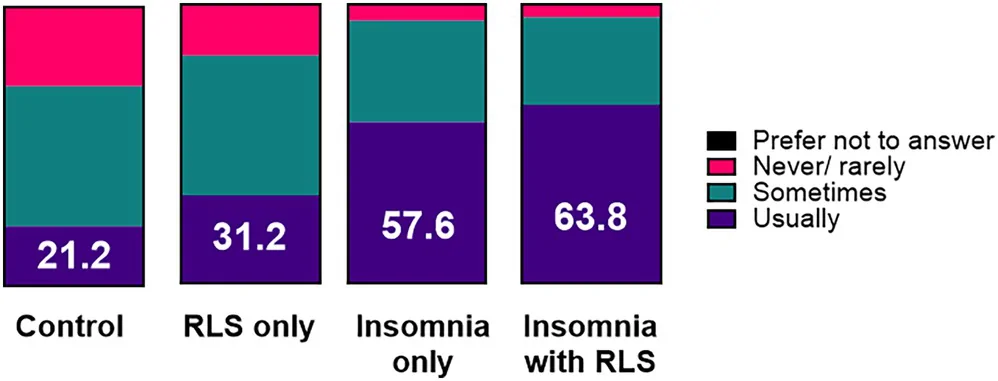
Response proportions to the single question used in prior insomnia GWAS. Responses are shown as percentages for each instrumentally‐defined group using the expanded sleep questionnaire in the UK Biobank. Prior GWAS for insomnia require a response of ‘usually’ (purple) to the question ‘Do you have trouble falling asleep at night or do you wake up in the middle of the night’? for inclusion into the insomnia case definition.

### 3.3 Examining SNP Effects on Instrumentally‐Defined Disorders

To assess the contribution of each single nucleotide polymorphism (SNP) to insomnia and RLS defined using validated instruments (Jansen et al. 2019; El Gewely et al. 2018; Schormair et al. 2024; Edelson et al. 2023), we first employed multinomial logistic regression models accounting for technical and demographic covariates (see Section 2). In the fully adjusted model (Model 2), we found the minor allele for the *BTBD9* SNP rs61192259(C) was significantly associated with a reduction in the likelihood of RLS (OR [95% CI]: 0.83 [0.80, 0.86], *p* < 2.2 x 10^−16^) and insomnia with RLS (OR [95% CI]: 0.84 [0.80, 0.88], *p* = 5.3 x 10^−15^) (Table 1). However, no significant association was observed with insomnia only (OR [95% CI]: 1.00 [0.98, 1.02], *p* = 0.85) (Table 1), indicating the *BTBD9* SNP is not associated with clinically relevant insomnia in the absence of RLS. The significant associations remained when RLS status was further refined by severity into intermittent (≤ 1*x* per week) and persistent RLS (> 1*x* per week) (Table S6), validating this SNP as a putative RLS risk variant.

**TABLE 1:** Multinomial logistic regression testing variant effects on categorical disease status.

SNP	Model	Group	MAF	OR (95% CI)	*p*
*BTBD9* rs61192259(C)	Model 1	Insomnia only	0.401	1.01 (0.99, 1.02)	0.58
		RLS only	0.356	0.83 (0.80, 0.86)	< 2.2 × 10^−16^
		Insomnia with RLS	0.359	0.84 (0.80, 0.88)	1.4 × 10^−14^
	Model 2	Insomnia only	0.401	1.00 (0.98, 1.02)	0.85
		RLS only	0.356	0.83 (0.80, 0.86)	< 2.2 × 10^−16^
		Insomnia with RLS	0.359	0.84 (0.80, 0.88)	5.3 × 10^−15^
*MEIS1* rs113851554(T)	Model 1	Insomnia only	0.051	1.06 (1.02, 1.11)	0.005
		RLS only	0.086	1.84 (1.73, 1.96)	< 2.2 × 10^−16^
		Insomnia with RLS	0.084	1.80 (1.66, 1.95)	< 2.2 x 10^−16^
	Model 2	Insomnia only	0.051	1.07 (1.02, 1.11)	0.003
		RLS only	0.086	1.85 (1.74, 1.98)	< 2.2 × 10^−16^
		Insomnia with RLS	0.084	1.81 (1.67, 1.96)	< 2.2 × 10^−16^

*Note:* Variant effects are presented as an odds ratio (OR) and 95% confidence interval (CI) for the minor allele for rs61192259(C) and rs113851554(T). Model 1 includes genotype array and the first 10 ancestry principal components as covariates. Model 2 includes age, sex and BMI in addition to Model 1's covariates.

Abbreviation: MAF, minor allele frequency.

The minor allele for the *MEIS1* SNP, rs113851554(T), showed a strong association with increased risk for RLS (OR [95% CI]: 1.85 [1.74, 1.98], *p* < 2.2 × 10^−16^) and insomnia with RLS (OR [95% CI]: 1.81 [1.67, 1.96], *p* < 2.2 × 10^−16^) (Table 1). Contrary to the *BTBD9* SNP, the *MEIS1* SNP remained significantly associated with the ‘insomnia only’ group (OR [95% CI]: 1.07 [1.02, 1.11], *p* = 0.003). However, the association with insomnia is markedly weaker than that with RLS. When the RLS phenotype was refined by frequency, the *MEIS1* SNP was associated with an elevated risk for both persistent RLS (OR [95% CI]: 1.92 [1.75, 2.10], *p* < 2.2 × 10^−16^) and intermittent RLS (OR [95% CI]: 1.81 [1.66, 1.98], *p* < 2.2 × 10^−16^) (Table S6).

It has been argued that *MEIS1* exhibits a pleiotropic effect on insomnia and RLS (Watanabe et al. 2022; El Gewely et al. 2018), in that it influences the pathogenesis of both traits independently. To better address the potential for pleiotropy between RLS and insomnia at the *MEIS1* SNP, we ran a logistic regression model with and without adjustment for RLS severity. Consistent with the multinomial model, the *MEIS1* SNP was associated with increased risk for insomnia after controlling for age, sex, BMI, genotyping array and ancestry informative principal components (Model 2 OR [95% CI]: 1.09 [1.05, 1.14], *p* = 5.3 × 10^−6^). This association was attenuated, but remained statistically significant after further adjustment for RLS (Model 3 OR [95% CI]: 1.05 [1.01, 1.09], *p* = 0.01) (Table 2). These results indicate that the *MEIS1* SNP maintains an association with insomnia risk that is independent of underlying RLS.

**TABLE 2:** Logistic regression testing SNP effects on insomnia controlling for RLS severity.

SNP	Model	OR (95% CI)	*p*
*BTBD9* rs61192259(C)	Model 1	1.00 (0.98–1.01)	0.55
	Model 2	0.99 (0.98–1.01)	0.42
	Model 3	1.00 (0.98–1.02)	0.83
*MEIS1* rs113851554(T)	Model 1	1.10 (1.06–1.14)	2.50 × 10^−6^
	Model 2	1.09 (1.05–1.14)	5.33 × 10^−6^
	Model 3	1.05 (1.01–1.09)	0.01

*Note:* Odds ratio (OR) and 95% confidence interval (CI) for the minor allele for rs61192259(C) and rs113851554(T). Model 1 includes genotype array and the first 10 ancestry principal components as covariates. Model 2 includes age, sex and BMI in addition to Model 1's covariates. Model 3 includes RLS severity in addition to Model 2's covariates.

The categorical definition used to define insomnia applies a threshold score of 16 out of 32 on the SCI (Espie et al. 2014), with a lower score indicating worse sleep quality. Provided this questionnaire captures clinically‐relevant sleep quality, a variant associated with sleep quality would be expected to show a linear relationship with the quantitative score. To test whether the *BTBD9* or *MEIS1* SNPs were associated with sleep quality measured by the SCI, we performed linear regression analyses using multiple models adjusting for covariates (Section 2). We found that each additional copy of the minor allele for the *MEIS1* SNP was associated with a statistically significant reduction in SCI score, including a 0.23‐point reduction for each copy of rs113851554(T) (*p* = 2.94 × 10^−4^) after accounting for RLS severity (Table 3). The *BTBD9* SNP did not significantly impact SCI score when controlling for RLS severity (Table 3). When RLS severity was not considered in the model (Model 2), the *BTBD9* SNP had a negligible effect on SCI score (β = 0.06, SE = 0.03, *p* = 0.03). Together, these analyses show that this *MEIS1* SNP is associated with sleep quality, even after accounting for clinically‐defined RLS, while the *BTBD9* SNP exerts its effect on RLS without a substantial impact on sleep quality.

**TABLE 3:** Linear regression testing variant effects on quantitative SCI score.

SNP	Model	Beta (SE)	*p*
*BTBD9* rs61192259(C)	Model 1	0.05 (0.03)	0.06
	Model 2	0.06 (0.03)	0.03
	Model 3	0.02 (0.03)	0.39
*MEIS1* rs113851554(T)	Model 1	−0.42 (0.07)	1.34 × 10^−10^
	Model 2	−0.40 (0.06)	4.46 × 10^−10^
	Model 3	−0.23 (0.06)	2.94 × 10^−4^

*Note:* Variant effects are presented as a Beta coefficient and standard error (SE for the minor allele for rs61192259(C) and rs113851554(T)). Model 1 includes genotype array and the first 10 principal components as covariates. Model 2 includes age, sex and BMI in addition to Model 1's covariates. Model 3 includes Model 2's covariates and additionally adds RLS severity as a covariate.

## 4 Discussion

GWAS reports for insomnia have historically been based on a single question (Watanabe et al. 2022; Lane et al. 2017; Jansen et al. 2019; Hammerschlag et al. 2017), resulting in a lack of specificity in the phenotypic definition and differential diagnosis from other sleep disturbances such as RLS. We integrated the new sleep questionnaires from the UK Biobank, which are based on diagnostic criteria and clinically‐validated instruments, to address potential confounding or pleiotropy for two of the most significant shared genome‐wide association signals for insomnia and RLS.

In multinomial regression analyses, the *MEIS1* locus showed strong associations with RLS‐containing phenotypes and only a modest, but statistically significant, association with insomnia both in the absence of RLS and when controlling for RLS severity as a covariate. This suggests that while the primary genetic effect likely operates through RLS risk, the *MEIS1* locus exerts a small residual influence on insomnia risk. Importantly, this association was not observed for the *BTBD9* locus, indicating previous GWAS for insomnia have likely misidentified *BTBD9* via its effect on RLS. *MEIS1* variants, however, may exhibit a pleiotropic effect on both disorders. Interestingly, linear regression models indicated the *MEIS1* risk allele reduces sleep quality as measured by the SCI even after accounting for RLS. However, the *BTBD9* locus had a negligible effect on sleep quality.

We also demonstrate that the single question typically used to phenotype insomnia in GWAS is not sufficient to differentiate RLS from insomnia. Moreover, this question misclassifies many individuals that should be considered controls, limiting its utility in identifying true genetic associations to the clinically‐defined disorder. The odds ratio (1.85) for the association between the *MEIS1* intronic variant, rs113851554, and RLS defined by the CH‐RLSq here is similar to previous GWAS reports (Schormair et al. 2024; Didriksen et al. 2020) demonstrating consistency in this questionnaire. However, the odds ratio for the association we observed between rs113851554 and insomnia after controlling for RLS is much lower than previous GWAS reporting at this variant (1.05 vs. 1.23) (Jansen et al. 2019), indicating the likely presence of unaccounted RLS in previous GWAS. Future GWAS for insomnia using validated quantitative questionnaires will better define insomnia and refine genetic risk factors.

We defined insomnia and RLS using the suggested thresholds and inclusion criteria from respective questionnaires, which resulted in a prevalence of each disorder consistent with previous literature (Ohayon et al. 2012; Roth 2007). However, insomnia is a heterogeneous disorder, and further stringency may be placed on the definitions of these disorders to define chronicity or severity. We also included covariates that are regularly used in GWAS for insomnia (Watanabe et al. 2022; Lane et al. 2017; Jansen et al. 2019; Hammerschlag et al. 2017) to allow for direct comparison. However, future work may address other correlated outcomes. Ultimately, our work highlights the utility of phenotyping within the UK Biobank that is based on validated instruments and provides a basis for improving future genomic studies that rely on these datasets.

## Author Contributions

**Amber J. Zimmerman:** formal analysis, writing – original draft, writing – review and editing, visualization, conceptualization. **Patrick Z. Liu:** methodology, writing – review and editing, formal analysis, data curation. **Brendan T. Keenan:** writing – review and editing, methodology, conceptualization, data curation. **Thomas G. Brooks:** writing – review and editing, formal analysis. **Struan F. A. Grant:** supervision, writing – review and editing. **Philip R. Gehrman:** writing – review and editing, supervision. **Ron C. Anafi:** writing – review and editing, supervision, resources. **Allan I. Pack:** resources, supervision, conceptualization, writing – review and editing, funding acquisition.

## Funding

This work was supported by National Heart, Lung, and Blood Institute (T32HL07953, P01HL160471) and National Institute on Aging (R01AG068577).

## Conflicts of Interest

The authors declare no conflicts of interest.

## Supporting information


**Table S1:** Total number of participants and filtering for exclusions. Total remaining indicates number of participants left after previous filtering step. Exclusions indicate the number of participants excluded in each category.
**Table S2:** Categorical definitions based on questionnaires. Sleep Condition Indicator (SCI) was used to define clinically‐relevant insomnia with a threshold of ≤ 16. RLS was defined by the CH‐RLSq. Intermittent RLS was defined by the CH‐RLSq with a frequency ≤ 1*x* per week. Persistent RLS was defined as a frequency > 1*x* per week.
Supporting Information **Table S3:** Responses for single question used in prior insomnia GWAS reports.

**Table S1:** Sleep Condition Indicator (SCI) questionnaire for defining insomnia. A score of ≤ 16 out of 32 was used to characterise insomnia. Questions and response options are provided along with the corresponding UK Biobank ID.
**Table S2:** Cambridge–Hopkins RLS questionnaire (CH‐RLSq) for defining RLS. Questions are provided as they appear in the UKBB questionnaire. The corresponding UK Biobank IDs are provided. The possible responses are provided for each question along with how they were assigned in the model. In models when RLS only is listed, no specific frequency was required. The only responses excluded included ‘never’ and ‘do not know’. In models subdividing RLS, intermittent RLS and persistent RLS are characterised by frequency of ≤1 day/week or > 1 day per week, respectively. Question 7a from the CHRLSq was not included in the UK Biobank.
**Table S3:** Demographics by instrumentally‐defined group (4 groups). Criteria for grouping are as follows: controls (SCI > 16 and no RLS), isolated RLS or ‘RLS only’ (SCI > 16 and RLS with no frequency threshold), isolated insomnia or ‘Insomnia only’ (SCI≤16 and no RLS), and ‘Insomnia with RLS’ (SCI ≤ 16 and RLS with no frequency threshold). Data presented as mean (SD) or percent.
**Table S4:** Demographics by instrumentally‐defined group refined by RLS severity. Criteria for grouping are as follows: controls (SCI > 16 and no RLS), isolated insomnia or ‘insomnia only’ (SCI ≤16 and no RLS of either type), ‘insomnia with RLS’ (SCI > 16 and RLS of either type), ‘intermittent RLS’ (RLS frequency ≤1 day per week) and ‘persistent RLS’ (RLS frequency > 1 day per week).
**Table S5:** Response proportions for insomnia GWAS question. Question 1200 from the UK Biobank used in previous GWAS reports for insomnia asks participants ‘Do you have trouble falling asleep at night or do you wake up in the middle of the night’? Response proportions are provided as percentages for each instrumentally‐defined category based on the sleep questionnaire data in the UK Biobank.
**Table S6:** Multinomial logistic regression testing variant effects on categorical disease status with refined RLS types. Variant effects are presented as an odds ratio (OR) and 95% confidence interval (CI) for the minor allele for rs61192259(C) and rs113851554(T). Model 1 includes genotype array and the first 10 ancestry principal components as covariates. Model 2 includes age, sex, and BMI in addition to Model 1's covariates. MAF = minor allele frequency. Results with a *p‐*value < 0.05 are bolded.

## Data Availability

The data that supports the findings of this study are available in the supplementary material of this article.
