# Supplementary material for: Phenotype Refinement Influences GWAS‐Implicated Variant Effect Sizes for Insomnia
Source: J Sleep Res. 2026 May 26;35(5):e70356. doi: 10.1111/jsr.70356 (PMC13619021; doi:10.1111/jsr.70356)
Supplement: Supplementary file 1 [file JSR-35-e70356-s001.docx]

**Supplementary Tables 1-6**

| **SCI question** | **Score = 4** | **Score = 3** | **Score = 2** | **Score = 1** | **Score = 0** | **UKBB ID** |
| --- | --- | --- | --- | --- | --- | --- |
| **Thinking about a typical night in the last month …** |  | | | | | |
| 1. How long does it take you to fall asleep? | 0–15 min | 16–30 min | 31–45 min | 46–60 min | >61 min | 30474 |
| 2. If you then wake up during the night… how long are you awake for in total? | 0–15 min | 16–30 min | 31–45 min | 46–60 min | >61 min | 30536 |
| 3. How many nights a week do you have a problem with your sleep? | 0–1 | 2 | 3 | 4 | 5–7 | 30538 |
| 4. How would you rate your sleep quality? | Very good | Good | Average | Poor | Very poor | 30539 |
| **Thinking about the past month, to what extent has poor sleep …** |  | | | | | |
| 5. … affected your mood, energy, or relationships? | Not at all | A little | Somewhat | Much | Very much | 30540 |
| 6. … affected your concentration, productivity, or ability to stay awake? | Not at all | A little | Somewhat | Much | Very much | 30541 |
| 7. … troubled you in general? | Not at all | A little | Somewhat | Much | Very much | 30542 |
| **Finally …** |  |  |  |  |  |  |
| 8. How long have you had a problem with your sleep? | I don’t have a problem / <1 mo | 1–2 mo | 3–6 mo | 7–12 mo | >1 yr | 30543 |

**Supplementary Table 1.** Sleep Condition Indicator (SCI) questionnaire for defining insomnia. A score of ≤ 16 out of 32 was used to characterize insomnia. Questions and response options are provided along with the corresponding UK Biobank ID.

| **Question #**  **in CH-RLSq** | **Question from CH-RLSq** | **UKBB ID** | **Response Scoring** | **Intermittent/Any RLS** | **Persistent RLS** |
| --- | --- | --- | --- | --- | --- |
| 1 | Do you have, or have you had, recurrent uncomfortable feelings or sensations in your legs while you are sitting or lying down? | 30579 | (1 = yes, 0 = no, -1 = do not know) | Yes | Yes |
| 2 | Do you, or have you had, a recurrent need or urge to move your legs while you were sitting or lying down? | 30580 | (1 = yes, 0 = no, -1 = do not know) | Yes | Yes |
| 3 | Are you more likely to have these feelings when you are resting (either sitting or lying down) or when you are physically active? | 30581 | (-1 = do not know, 1 = resting, 2 = active) | Resting | Resting |
| 4 | If you get up or move around when you have these feelings do these feelings get any better while you actually keep moving? | 30582 | (1 = yes, 0 = no, -1 = do not know) | Yes | Yes |
| 5 | Which times of day are these feelings in your legs most likely to occur? (Please circle one or more than one) | 30583 | (-1 = do not know, 1 = morning, 2 = midday, 3 = afternoon, 4 = evening, 5= night, 6 = about equal at all times) | NOT equal or morning | NOT equal or morning |
| 6 | Will simply changing leg position by itself once without continuing to move usually relieve these feelings? | 30584 | (-1 = do not know, 1 = usually relieves, 2 = does not usually relieve) | Does not usually relieve | Does not usually relieve |
| 7a. | Are these feelings ever due to muscle cramps? | NA |  | No | No |
| 7b. | If so, are they always due to muscle cramps? | 30585 | (1 = yes, 0 = no, -1 = do not know) | No | No |
| NA | In the past 12 months, how often did you experience these feelings in your legs? | 32070 | Frequency of feelings in legs in past 12 months (-1 = do not know, 0 = never, 1 = 6-7 days/week, 2 = 4-5d/wk, 3 = 2-3d/wk, 4 = 1d/wk, 5 = 2d/month, 6 = 1d/month or less) | Any answer other than “never” or “do not know” was used in models when RLS is not subdivided.  Intermittent RLS = 1d/wk, 2d/month, or 1d/month or less | 6-7 days/week OR 4-5 days/week OR 2-3 days/week |

**Supplementary Table 2.** Cambridge-Hopkins RLS questionnaire (CH-RLSq) for defining RLS. Questions are provided as they appear in the UKBB questionnaire. The corresponding UK Biobank IDs are provided. The possible responses are provided for each question along with how they were assigned in the model. In models when RLS only is listed, no specific frequency was required. The only responses excluded included “never” and “do not know”. In models subdividing RLS, intermittent RLS and persistent RLS are characterized by frequency of $\leq$1 day/ week or >1 day per week, respectively. Question 7a from the CHRLSq was not included in the UK Biobank.

| **Category** | **Control** | **RLS only** | **Insomnia only** | **Insomnia with RLS** |
| --- | --- | --- | --- | --- |
| Age (years) | 70.5 (7.4) | 69.8 (7.3) | 69.8 (7.5) | 69.5 (7.4) |
| BMI (kg/m^2^) | 26.5 (4.4) | 26.5 (4.6) | 27.1 (4.9) | 27.1 (5.0) |
| Female | 53.0% | 70.7% | 67.9% | 79.3% |

**Supplementary Table 3. Demographics by instrumentally-defined group (4 groups).** Criteria for grouping are as follows: controls (SCI>16 and no RLS), isolated RLS or “RLS only” (SCI>16 and RLS with no frequency threshold), isolated insomnia or “Insomnia only” (SCI≤16 and no RLS), and “Insomnia with RLS” (SCI≤16 and RLS with no frequency threshold). Data presented as mean (SD) or percent.

| **Category** | **Control** | **Insomnia only** | **Insomnia with RLS** | **Intermittent RLS** | **Persistent RLS** |
| --- | --- | --- | --- | --- | --- |
| age [years: mean (SD)] | 70.5 (7.41) | 69.8 (7.52) | 69.5 (7.35) | 69.5 (7.22) | 70.2 (7.40) |
| BMI | 26.5 (4.35) | 27.1 (4.90) | 27.1 (5.04) | 26.1 (4.49) | 26.9 (4.78) |
| %Female | 53 | 67.9 | 79.3 | 72.3 | 68.9 |

**Supplementary Table 4. Demographics by instrumentally-defined group refined by RLS severity**. Criteria for grouping are as follows: controls (SCI > 16 and no RLS), isolated insomnia or “insomnia only” (SCI ≤ 16 and no RLS of either type), “insomnia with RLS” (SCI > 16 and RLS of either type), “intermittent RLS” (RLS frequency $\leq$1 day per week) and “persistent RLS” (RLS frequency >1 day per week).

| **Response to Question 1200** | **Control (%)** | **RLS only (%)** | **Insomnia with RLS (%)** | **Insomnia only (%)** |
| --- | --- | --- | --- | --- |
| Prefer not to answer | 0.06 | 0.02 | 0 | 0.04 |
| Never/ rarely | 28.43 | 18.54 | 4.71 | 5.96 |
| Sometimes | 50.31 | 50.2 | 31.45 | 36.44 |
| Usually | 21.19 | 31.24 | 63.84 | 57.56 |

**Supplementary Table 5. Response proportions for insomnia GWAS question.** Question 1200 from the UK Biobank used in previous GWAS reports for insomnia asks participants “Do you have trouble falling asleep at night or do you wake up in the middle of the night?”. Response proportions are provided as percentages for each instrumentally-defined category based on the sleep questionnaire data in the UK Biobank.

| **SNP** | **Model** | **Group** | **MAF** | **OR (95% CI)** | ***p*-value** |
| --- | --- | --- | --- | --- | --- |
| *BTBD9*  rs61192259(C) | Model 1 | Insomnia only | 0.401 | 1.00 (0.99, 1.02) | 0.61 |
|  |  | Insomnia with RLS | 0.359 | 0.84 (0.80, 0.88) | **3.55×10^−15^** |
|  |  | Intermittent RLS | 0.352 | 0.82 (0.78, 0.86) | **1.78×10^−15^** |
|  |  | Persistent RLS | 0.36 | 0.85 (0.81, 0.89) | **7.97×10^−10^** |
|  | Model 2 | Insomnia only | 0.401 | 1.00 (0.98, 1.02) | 0.86 |
|  |  | Insomnia with RLS | 0.359 | 0.83 (0.79, 0.87) | **4.44×10^−16^** |
|  |  | Intermittent RLS | 0.352 | 0.80 (0.77, 0.85) | **<2.2×10^−16^** |
|  |  | Persistent RLS | 0.36 | 0.84 (0.79, 0.88) | **1.08×10^−10^** |
| *MEIS1*  rs113851554(T) | Model 1 | Insomnia only | 0.051 | 1.06 (1.02, 1.10) | **0.007** |
|  |  | Insomnia with RLS | 0.084 | 1.79 (1.66, 1.93) | **<2.2×10^−16^** |
|  |  | Intermittent RLS | 0.084 | 1.76 (1.61, 1.92) | **<2.2×10^−16^** |
|  |  | Persistent RLS | 0.088 | 1.87 (1.70, 2.04) | **<2.2×10^−16^** |
|  | Model 2 | Insomnia only | 0.051 | 1.07 (1.02, 1.11) | **0.003** |
|  |  | Insomnia with RLS | 0.084 | 1.81 (1.68, 1.96) | **<2.2×10^−16^** |
|  |  | Intermittent RLS | 0.084 | 1.81 (1.66, 1.98) | **<2.2×10^−16^** |
|  |  | Persistent RLS | 0.088 | 1.92 (1.75, 2.10) | **<2.2×10^−16^** |

**Supplementary Table 6. Multinomial logistic regression testing variant effects on categorical disease status with refined RLS types.** Variant effects are presented as an odds ratio (OR) and 95% confidence interval (CI) for the minor allele for rs61192259(C) and rs113851554(T). Model 1 includes genotype array and the first 10 ancestry principal components as covariates. Model 2 includes age, sex, and BMI in addition to Model 1’s covariates. MAF = minor allele frequency. Results with a *p-*value < 0.05 are bolded.
